# Water Extract of *Ecklonia cava* Protects against Fine Dust (PM_2.5_)-Induced Health Damage by Regulating Gut Health

**DOI:** 10.4014/jmb.2203.03020

**Published:** 2022-06-06

**Authors:** Seon Kyeong Park, Jin Yong Kang, Jong Min Kim, Min Ji Kim, Hyo Lim Lee, Jong Hyun Moon, Hye Rin Jeong, Hyun-Jin Kim, Ho Jin Heo

**Affiliations:** 1 Division of Applied Life Science (BK21), Institute of Agriculture and Life Science, Gyeongsang National University, Jinju 52828, Republic of Korea; 2 Korea Food Research Institute, Jeollabuk-do 55365, Republic of Korea; 3 Fermentation Regulation Research Group, World Institute of Kimchi, Gwangju 61755, Republic of Korea

**Keywords:** *Ecklonia cava*, microbiota, gut-brain axis, short-chain fatty acid, kynurenine pathway, PM_2.5_

## Abstract

To confirm the therapeutic effect of the water extract from *Ecklonia cava* (WEE) against PM_2.5_ induced systemic health damage, we evaluated gut health with a focus on the microbiota and metabolites. Systemic damage in mice was induced through PM_2.5_ exposure for 12 weeks in a whole-body chamber. After exposure for 12 weeks, body weight and food intake decreased, and WEE at 200 mg/kg body weight (mpk) alleviated these metabolic efficiency changes. In addition, PM_2.5_ induced changes in the length of the colon and fecal water content. The administration of the WEE at 200 mpk oral dose effectively reduced changes in the colon caused by PM_2.5_ exposure. We also attempted to confirm whether the effect of the WEE is mediated via regulation of the microbiota-gut-brain axis in mice with PM_2.5_ induced systemic damage. We examined changes in the fecal microbiota and gut metabolites such as short-chain fatty acids (SCFAs) and kynurenine metabolites. In the PM_2.5_ exposed group, a decrease in the abundance of *Lactobacillus* (Family: *Lactobacillaceae*) and an increase in the abundance of *Alistipes* (Family: *Rikenellaceae*) were observed, and the administration of the WEE showed a beneficial effect on the gut microbiota. In addition, the WEE effectively increased the levels of SCFAs (acetate, propionate, and butyrate). Furthermore, kynurenic acid (KYNA), which is a critical neuroprotective metabolite in the gut-brain axis, was increased by the administration of the WEE. Our findings suggest that the WEE could be used as a potential therapeutic against PM_2.5_ induced health damage by regulating gut function.

## Introduction

Over the past 10 years, air pollution has been reported to have a marked impact on public health [1]. In particular, fine particulate matter (PM_2.5_) with an aerodynamic diameter ≤ 2.5 μm is classified as a serious health hazard [2]. Recent studies have shown a strong association between PM_2.5_ exposure and the incidence of cognitive dysfunction [3, 4]. Some studies reported the relationship of PM_2.5_ exposure with various gastrointestinal diseases such as inflammatory bowel disease, appendicitis, and irritable bowel syndrome [5, 6]. PM_2.5_ exposure alters the gut microbiota and induces changes in epithelial tight junctions with ROS production and pro-inflammatory responses [5, 7]. Alternations in the gut microbiota can lead to impaired gut permeability and cause systemic inflammation and central nervous system diseases [8].

Recent studies have demonstrated a link between the microbiota and cognitive function [9]. The gut microbiota is known to affect brain function through the vagus nerve and the production of metabolites such as short-chain fatty acids (SCFAs), cytokines, and tryptophan, kynurenine [10]. Microbiota dysbiosis is associated with altered immunity, leading to systemic disorders [11]. In particular, the kynurenine pathway has been reported as a critical pathway involved in various central nervous system and gastrointestinal activities, and its association with mental diseases such as schizophrenia, depression, and dementia has been suggested [8]. The kynurenine pathway is initiated by the metabolism of the essential amino acid tryptophan in the gut [12]. Metabolites produced by the kynurenine pathway are key regulators in maintaining neuronal cell function. For example, kynurenic acid (KYNA), picolinic acid, and the essential cofactor NAD+ are associated with a neuroprotective effect, whereas quinolinic acid (QUIN) and 3-hydroxykynurenine have a neurotoxic effect [13]. Therefore, regulation of the kynurenine pathway through the gut-brain axis may be an interesting target for therapeutics to improve cognitive function [8].

*Ecklonia cava* (*E. cava*, Korean: Gamtae) is a species endemic to the temperate waters of Korea and is mainly found around the Jeju and Dokdo Islands [14]. The various health benefits of *E. cava* (*e.g.*, antioxidant, anti-inflammation, anti-diabetic, cognitive improvement, and anti-obesity effects) have been reported recently [15, 16] . In our previous study, it was found that the water extract from *Ecklonia cava* (WEE) effectively prevented PM_2.5_-induced cognitive decline, and the major bioactive compounds of the WEE were fucoidan and phlorotannins [17]. However, it has been reported that fucoidan and most phlorotannins do not directly affect brain function as they cannot pass through the blood-brain-barrier (B-B-B) [18-20]. On the other hand, metabolites produced by intestinal fermentation have been reported to affect cognitive function. Thus far, a gut health-related study of *E. cava* has not been conducted. Therefore, it is necessary to investigate the correlation between the gut and brain tissue according to WEE intake. Our study aimed to determine whether the effect of the WEE is mediated via regulation of the microbiota and metabolites in a PM_2.5_ exposed mouse model.

## Materials and Methods

### Preparation of Water Extract from *E. cava*

*E. cava* was purchased from Parajeju (Korea) in February 2018. Powdered *E. cava* (10 g) was extracted with distilled water (2 L) at 40 °C for 2 h. The water extract from *E. cava* was filtered under vacuum using Whatman No. 2 filter paper (Whatman International Limited, UK), and lyophilized using a vacuum-tray freeze dryer (Operon, Korea). Lyophilized extract was stored at −20°C.

### Experimental Design

All animal experimental protocols were approved by the Institutional Animal Care and Use Committee of Gyeongsang National University (certificate: GNU-180927-M0050, 27 September 2018) and conducted in accordance with the Policy of the Ethical Committee of the Ministry of Health and Welfare in Korea. BALB/c mice (male, 6 weeks) were obtained from Samtako (Korea) and were randomly divided into 6 groups (*n* = 7). The animals were maintained with a 12 h light/dark cycle and 55% humidity at 22 ± 2°C. The groups consisted of a normal control (NC) group, a PM_2.5_-exposed group, and WEE groups (50, 100, and 200 mg/kg body weight; WEE50, WEE100, and WEE200, respectively). The WEE (dissolved drinking water) was orally administered using a stomach tube before PM_2.5_ exposure. Subsequently, the animals were exposed to PM_2.5_ (500 μg/m^3^) or filtered air in a whole-body exposure chamber for 5 h/day and 5 days/week for 12 weeks. Body weight and food intake were measured every 4 weeks [17].

### Fecal Water Content

After 12 weeks of WEE intake and PM_2.5_ exposure, feces were collected in a metabolic cage (Jeung Do Bio & Plant Co. Ltd., Korea). The fecal water content of a portion of the collected feces was immediately determined. The fecal water content was measured using an infrared moisture analyzer.

### Tissue Preparation

After PM_2.5_ exposure for 12 weeks, mice were sacrificed. The blood was collected from the abdominal aorta vein into a plasma heparin tube and centrifuged at 3,000 ×*g* for 10 min at 4°C. Brain and large intestine tissues were immediately removed. The removed tissues were immersed in cold PBS for 5 min and stored at −80°C.

### Gut microbiota Composition Analysis

To determine changes in the gut microbiota, DNA was extracted from frozen feces and analyzed by 16S rRNA next-generation sequencing (NGS) analysis. Total DNA was extracted using a FastDNA Spin kit (MP Biomedicals) and Authentication Kit (Promega). The 16S ribosomal RNA gene of the V3-V4 regions was amplified using fusion primers (341F; 5’-CTACGGGNGGCWGCAG-3’, 805R; 5’-GACTACHVGGGTATCTAATCC-3’) by PCR (95°C for 3 min, 95°C for 30 sec, 55°C for 30 sec, and extension at 72°C for 30 sec (25 cycles) and a final elongation at 72°C for 5 min). To identify the PCR reactions, 1% agarose gel electrophoresis and visualization under a Gel Doc system (BioRad, USA) were carried out, and CleanPCR (CleanNA) was used to purify the amplified products. The purified products were pooled together at equal concentrations and short fragments (non-target products) were removed with CleanNA. The quality and sizes of the products were evaluated using a Bioanalyzer 2100 (Agilent, USA) using a DNA 7500 chip. After pooling the mixed amplicons, the bioinformatics cloud platform of CJ Bioscience, Inc. (Korea) was used to perform sequencing with an Illumina MiSeq Sequencing system (Illumina, USA). The quality of raw reads was checked before the start of processing and Trimmomatic ver. 0.32 was used to filter low-quality (< Q25) reads. The fastq_mergepairs command of VSEARCH version 2.13.4 with default parameters was used to merge paired-end sequence data together after QC pass. Primers were then trimmed followed by the extraction of unique reads and clustering of redundant reads with VSEARCH. Taxonomic assignment was carried out using the EzBioCloud 16S rRNA database. The UCHIME algorithm and the non-chimeric 16S rRNA database from EzBioCloud were used to carry out reference-based chimeric detection for filtering of chimeric reads on reads with similarity exceeding 97%. Further analytics of alpha diversity indices, rarefaction curves, rank abundance curves, and others were performed in EzBioCloud 16S-based MTP, which is a CJ Bioscience’s bioinformatics cloud platform.

### Fecal SCFA Analysis

Frozen feces were homogenized using a bullet blender (Next Advance Inc., USA) with 5 mM NaOH and centrifuged at 12,000 ×*g* for 10 min. The supernatant was mixed with 2-ethylbutyric acid as an internal standard. The mixture was reacted with propanol/pyridine (3:2) and propyl chloroformate in the hood, which was kept open for 1 min. Then, the lid was closed, and the sample was vortexed and sonicated. SCFAs were extracted using hexane, and the extracts were analyzed by GC-MS analysis using the GC-MS-TQ 8030 Triple Quadrupole Gas Chromatograph Mass Spectrometer (Shimadzu, Japan), GC/MS-QP 2010 Plus (Shimadzu), and DB-5MS column (30 m × 0.25 mm, thickness 0.25 μm). The GC conditions were as follows: split ratio: 60:1, injection temperature: 200°C, column oven temperature: 60°C, and flow rate: 1.0 ml/min. The initial temperature was 40°C, which was maintained for 5 min and then allowed to reach 310°C at a rate of 10°C/min [21].

### Kynurenine Metabolite Analysis

Kynurenine metabolites were analyzed with the modified method of Virág and Wang [22, 23]. The standard compounds (tryptophan (PHR1176), L-kynurenine (K8625), kynurenic acid (K3375), and quinolinic acid (P63204)) were purchased from Sigma Aldrich (USA). The colon and brain tissues were homogenized using a bullet blender (Next Advance Inc.) with a D.W./MeOH/ACN (1:2:2). The homogenized tissue and blood plasma was mixed with trifluoroacetic acid and centrifuged at 20,000 ×*g* for 15 min. The supernatant lyophilized using a vacuum-tray freeze dryer (Operon). Powdered extract was dissolved with MeOH/ACN (1:1) and centrifuged at 20,000 ×*g* for 15 min. The supernatants were injected into an ultraperformance liquid chromatography/quadrupole time-of-flight tandem mass spectrometry system (UPLC-Q-TOF-MS, Vion, Waters Corp., USA). The extracted samples were separated on the Acquity UPLC BEH C18 Column (2.1 mm × 100 mm, 1.7 μm; Waters Corp.) equilibrated with water: acetonitrile (ACN) containing 0.1% formic acid. Elution was carried out at a flow rate of 0.35 ml/min for 12 min. Mass spectrometric detection was conducted by multiple reaction monitoring (MRM) with an electrospray ionization (ESI) source in the positive mode. The LC/MS conditions were as follows: ramp collision energy, 20–45V; oven temperature, 40°C; capillary voltage, 3kV; and mass range, 50–1500. The MRM conditions for the analytes are shown in Table 1.

### Statistical Analysis

Data are presented as the mean ± standard deviation of mean (SD), and significant differences were determined by analysis of variance (ANOVA) with Duncan’s post-hoc analysis (*p* < 0.05) using SAS software (version 9.4; SAS Institute, USA).

## Results

### Changes in Body Weight and Food Intake

**Fig. 1:**
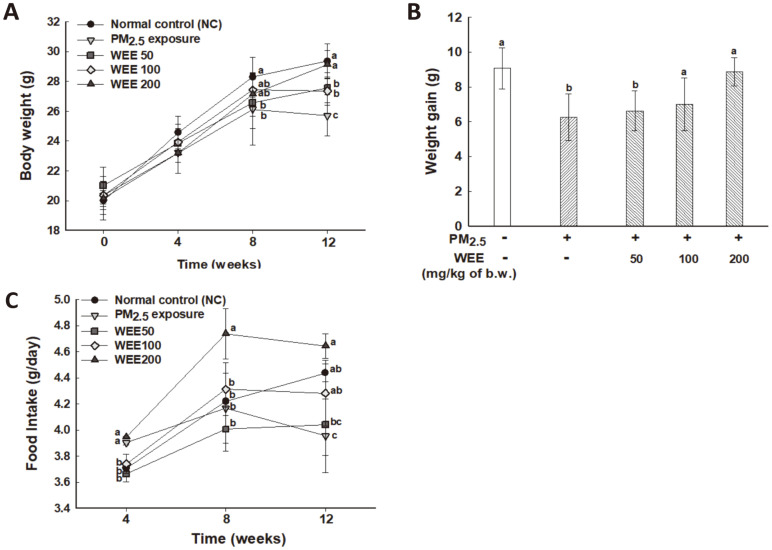
Effect of the water extract from *Ecklonia cava* (WEE) on fine dust (PM_2.5_)-exposed mice. Changes in body weight (**A**), weight gain (**B**), and food intake (**C**) for 12 weeks were determined. The results are presented as the mean ± SD (*n* = 7) and statistically significant at *p* < 0.05. Different lowercase letters indicate a statistical difference.

To investigate the toxicological response following PM_2.5_ exposure, we measured body weight and food intake once every 4 weeks for 12 weeks. Changes in body weight and food intake are shown in Fig. 1. The PM_2.5_ exposed group exhibited a significant difference in body weight from 8 week of exposure (Fig. 1A). At 12 weeks, the PM_2.5_ exposed group (25.71 g, weight gain of 6.25 g) showed a significant decrease in body weight compared with that of the NC group (29.36 g, weight gain of 9.06 g) (Figs. 1A and 1B). On the other hand, the WEE groups (WEE50: 27.57 g, weight gain of 6.63 g; WEE100: 27.36 g, weight gain of 7.00 g) showed an increasing trend in body weight with an increasing concentration of the WEE. In particular, WEE200 (29.14 g, weight gain of 8.88 g) showed a similar body weight compared with that of the NC group.

Changes in food intake are shown in Fig. 1C. The PM_2.5_ exposed group (3.96 g/day, around a decrease of 11%) showed a decrease in food intake compared with that of the NC group at 12 weeks. The WEE100 and WEE200 groups (4.28 and 4.64 g/day, respectively) showed a similar food intake compared with that of the NC group. Based on these results, WEE intake effectively prevented the PM_2.5_-induced decrease in metabolic efficiency.

### Changes in the Colon

**Fig. 2:**
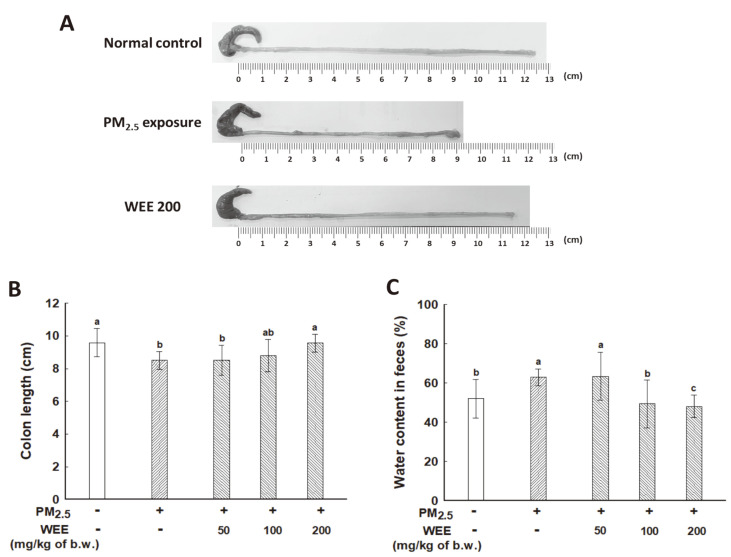
Changes in the colon and feces of fine dust (PM_2.5_)-exposed mice following the administration of the water extract from *Ecklonia cava* (WEE). Representative photographs of the colon (**A**), length of colon (**B**), and fecal water content (**C**). The results are presented as the mean ± SD (*n* = 7) and statistically significant at *p* < 0.05. Different lowercase letters indicate a statistical difference.

As shown in Fig. 2A, PM_2.5_ exposure resulted in changes in the colon. The PM_2.5_ exposed group (8.50 cm, around a decrease of 11%) showed a shortened colon length compared with that of the NC group (9.59 cm) (Fig. 2B). The WEE200 group (9.57 cm) showed a similar colon length compared with that of the NC group. To evaluate colon function, the fecal water content was measured. An increased fecal water content was observed in the PM_2.5_ exposed group (62.86%) compared with the NC group (52%) as shown in Fig. 2C. The effect of the WEE was significant starting at 100 mpk oral dose (WEE100: 49%; WEE200: 48%).

### Gut Microbiota Composition Analysis

**Fig. 3:**
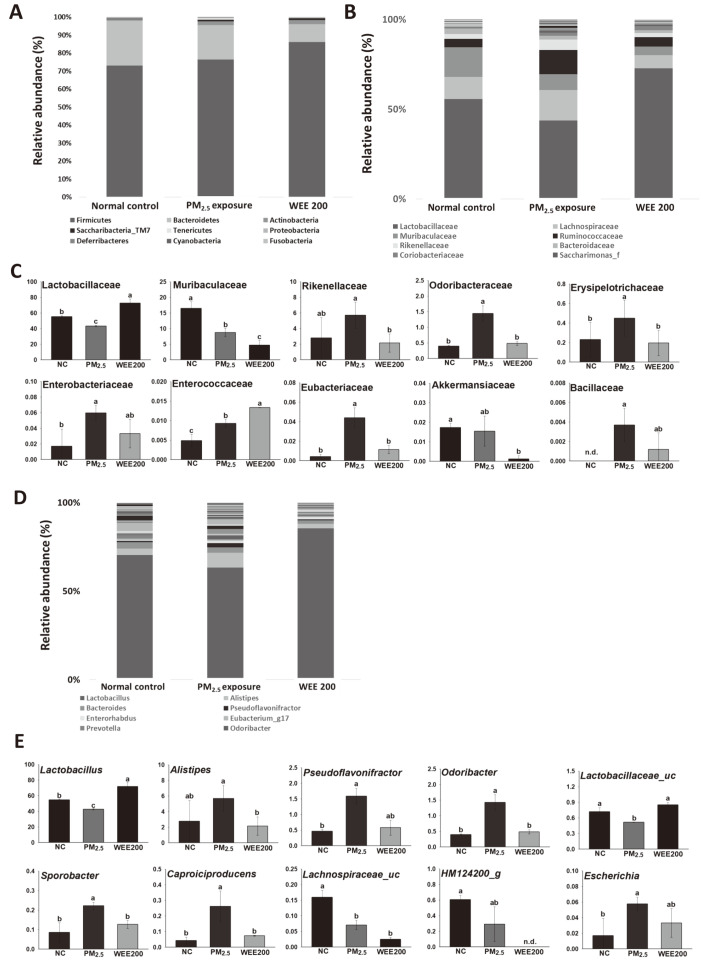
Changes in the gut microbiota of fine dust (PM_2.5_)-exposed mice following the administration of the water extract from *Ecklonia cava* (WEE). The relative abundances of the phylum (**A**), relative abundances of the family (**B**), significant changes of some families (**C**), relative abundances of the genus (**D**), and significant changes of some genera (**E**) of bacteria in feces were determined. The results are presented as the mean ± SD (*n* = 3) and statistically significant at *p* < 0.05. Different lowercase letters indicate a statistical difference.

There were no significant differences in bacterial phyla between the PM_2.5_ exposed and NC groups (Fig. 3A). The WEE200 group showed an increase in Firmicutes and a decrease in Bacteroidetes. Moreover, significant differences were observed at the family level (Figs. 3B and 3C). *Lactobacillaceae*, *Muribaculaceae*, and *Akkermanisiaceae* were less abundant, whereas *Rikenellaceae*, *Odoribacteraceae*, *Erysipelotrichaceae*, *Enterobacteriaceae*, *Enterococcaceae*, *Eubacteriaceae*, and *Bacillaceae* were more abundant in the PM_2.5_ exposed group compared with the NC group. The WEE200 group showed a restored gut microbiota at the family level. In particular, the abundance of *Lactobacillaceae*, the family of bacteria most affected by PM_2.5_ exposure (43.36% in the PM_2.5_-exposed group versus 55.44% in the NC group), was increased in the WEE200 group (72.63%). In addition, the relative abundance of *Rikenellaceae* was increased following PM_2.5_ exposure (5.70% in the PM_2.5_ exposed group versus 2.79% in the NC group), which was alleviated in the WEE200 group (2.16%). Furthermore, changes at the genus level demonstrated that the abundances of *Lactobacillus*, *Lactobacillaceae_uc*, *Lachnospiraceae_uc*, and *HM 124200_g* were decreased, whereas those of *Alistipes*, *Pseudoflavonifractor*, *Odoribacter*, *Sporobacter*, *Caproiciproducens*, and *Escherichia* were increased following PM_2.5_ exposure (Figs. 3D and 3E). The intake of the WEE restored the gut microbiota by increasing the amount of beneficial gut bacteria.

### Fecal SCFA Analysis

**Fig. 4:**
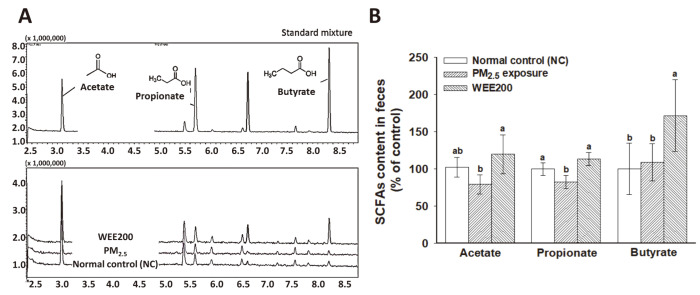
Analysis of the fecal short-chain fatty acids (SCFAs) of fine dust (PM_2.5_)-exposed BALB/c mice following the administration of the water extract from *Ecklonia cava* (WEE). GC/MS spectra of the standard mixture of each group (**A**) and SCFA contents in feces (**B**). The results are presented as the mean ± SD (*n* = 7) and statistically significant at *p* < 0.05. Different lowercase letters indicate a statistical difference.

Acetate (79.43%) and propionate (82.38%) levels were decreased in the PM_2.5_ exposed group, whereas the level of butyrate showed no statistical difference compared with that in the NC group (100.00%) (Figs. 4A and 4B). In comparison with the NC group, the WEE200 group exhibited increased SCFA production with a higher content of acetate (119.68%), propionate (113.30%), and butyrate (171.82%).

### Kynurenine Metabolite Analysis

**Fig. 5:**
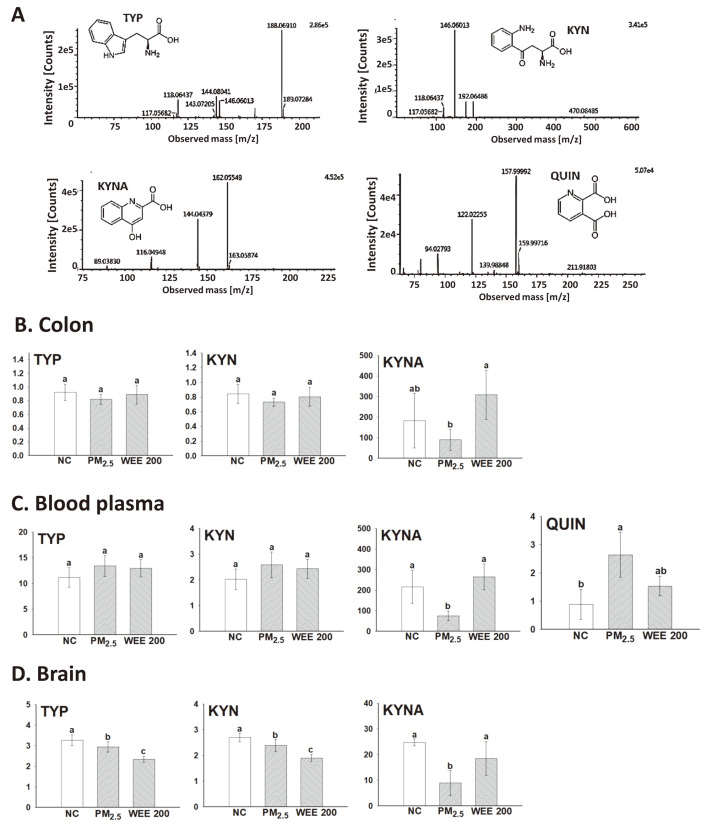
Analysis of kynurenine metabolites (tryptophan [TYP], kynurenine [KYN], kynurenic acid [KYNA], and quinolinic acid [QUIN]) using the UPLC/MS/MS system with multiple reaction monitoring (MRM). LC/MS^2^ fragment spectra of the standard mixture of each standard (**A**). The levels of kynurenine metabolites in the colon tissue (**B**), blood plasma (**C**), and brain tissue (**D**) were determined. The results are presented as the mean ± SD (*n* = 7) and statistically significant at *p* < 0.05. Different lowercase letters indicate a statistical difference.

Tryptophan (TYP) and kynurenine (KYN) levels in the colon showed no statistical difference between the groups. However, the level of KYNA was reduced in the PM_2.5_-exposed group compared with the NC group. In the WEE200 group, the level of KYNA was similar to that of the NC group (Figs. 5A and 5B). In the blood plasma, tryptophan and kynurenine levels also showed no statistical difference between the groups. However, the level of KYNA was reduced, and the level of QUIN was increased in BALB/c mice following PM_2.5_ exposure (Fig. 5C). The KYNA and QUIN levels of the WEE group were similar to those of the NC group. TYP and KYN levels in the colon were maintained in both the PM_2.5_ and WEE200 groups, while significantly decreased in the brain (Fig. 5D). QUIN was not detected, and the level of KYNA was reduced in the PM_2.5_-exposed group compared with the NC group. The administration of the WEE (200 mpk) maintained KYNA at a level similar to that of the NC group.

## Discussion

Recently, PM_2.5_ is considered as a serious health hazard worldwide [24]. Nevertheless, there are limited studies on PM_2.5_ related disease prevention. Previously, we identified fucoidan and phlorotannin as the major bioactive compounds in the WEE, and the WEE effectively prevented cognitive decline caused by PM_2.5_ [17]. However, molecules over 500 Da cannot pass through the tight junctions and B-B-B; thus, it is difficult to directly demonstrate their efficacy in treatment for neurodegenerative diseases [18, 19]. Fucoidan, which is a class of fucose-rich sulfated homo/heteropolysaccharides of 100–1,600 kDa, cannot pass through the B-B-B. In addition, most phlorotannins except eckol cannot pass through the B-B-B [18, 20]. Therefore, the enteric fermentation process may be required to affect the brain. We investigated the mechanism of *E. cava* as a therapeutic against PM_2.5_ induced cognitive dysfunction by evaluating the microbiota and associated metabolites.

In this study, our results demonstrated a decrease in body weight and food intake following PM_2.5_ exposure, and the administration of the WEE effectively maintained metabolic efficiency similar to that in the NC group (Fig. 1). Body weight is dependent on the balance between food intake and energy expenditure, and food intake affects the central nervous system through the microbiota-gut-brain crosstalk [25, 26]. In particular, gut hormones (*e.g.*, cholecystokinin, peptide YY, and oxyntomodulin) could inhibit food intake [25]. Fucoidan is known as a prebiotic for gut health, which is fermented by intestinal microbiota such as *Lactobacillus* strains in humans [20]. In addition, this prebiotic has been associated with brain function with either a direct or indirect effect on signaling molecules by producing gut hormones through neuropeptides (*e.g.*, peptide YY; PYY). Based on the results, the administration of the WEE (200 mpk oral dose) affected the gut-brain crosstalk in PM_2.5_ exposed mice.

The association between PM_2.5_ and gut function is still unclear. In a dextran sulfate sodium-induced inflammatory bowel disease model, intestinal barrier damage and inflammatory responses in the colon affected the length of the colon with morphological and histopathological changes [27]. It is well known that inflammation of the colon could result in edema of the colon, thus decreasing the length of the whole colon [28]. Our study confirmed functional changes in the gut following PM_2.5_ exposure by measuring the length of the colon and fecal water content. The results revealed changes in the colon length and fecal water content following PM_2.5_ exposure, and the WEE effectively prevented these changes and gut damage (Fig. 2). Inflammatory responses induce intestinal barrier dysfunction by decreasing the expression of tight junction proteins (*e.g.*, claudin-1, occludin, and ZO-1) [29]. Gut damage results in loose feces due to impaired water absorption in the colon. Fucoidan intake has been found to restore the microbiota and intestinal barrier function by regulating tight junction-associated proteins (ZO-1, occludin, claudin-1, and claudin-8) and the MAPK signaling pathway in a 7,12-dimethylbenz[a]anthracene (DMBA)-induced breast cancer rat model [30]. In addition, fucoidan has been reported to effectively maintain the intestinal barrier function by increasing ZO-1, claudin-1, and occludin expression in a non-obese diabetic mouse model [31]. These findings suggest that fucoidan could potentially modulate intestinal function by regulating the expression levels of tight junction-mediated proteins, which could alleviate changes in the length of the colon and fecal water content in the PM_2.5_-exposed mouse model resulting from intestinal damage and dysfunction. The WEE including fucoidan as a major bioactive compound may be a potential therapeutic for gut health.

The gut microbiota is a huge, complex ecosystem, and modulates the immunity and defense metabolism of the host [32]. Dysbiosis, the collapse of balance in the gut microbiota, has been considered to play an essential role in the increased risk of disease, and is closely associated with the regulation of oxidative stress and inflammatory response. Some recent studies reported that PM_2.5_ significantly altered the gut microbiota [6, 33]. At the genus level, the abundance of *Lactobacillus* was decreased, and *Escherichia*, *Parabacteroides*, *Akkermansia*, and *Oscillibacter* was significantly increased by PM_2.5_ exposure for 14 days [6]. Our study (Fig. 3) was limited in that it analyzed the composition of the microbiota with n=3/group. However, a decrease in *Lactobacillus*, *Lactobacillaceae_uc*, *Lachnospiraceae_uc*, and *HM 124200_g* and an increase in *Alistipes*, *Pseudoflavonifractor*, and *Odoribacter* following PM_2.5_ exposure for 12 weeks were observed at the genus level, and the administration of WEE regulated changes in the gut microbiota. Many studies have demonstrated that probiotics have a beneficial effect on health with the balanced regulation of pro-inflammatory (*i.e.*, IFN-γ and TNF-α) and anti-inflammatory (*i.e.*, IL-10 and IL-4) cytokines [34]. In particular, *Lactobacillus* spp. plays an essential role in modulating oxidative stress and inflammation with the regulation of the thioredoxin antioxidant system, Nrf-2 and NF-κB transcription factor-related mechanism [35]. These reports suggest that *Lactobacillus* spp. or prebiotics used to increase *Lactobacillus* spp. could potentially be applied for the prevention and early treatment of diseases. Therefore, WEE is able to improve intestinal function as well as have various health beneficial effects by increasing the abundance of *Lactobacillus* spp.

Thus, the gut is also known as the second brain, and the gut-brain axis has been an area of focus in the treatment of neurodegeneration [36, 37]. Recently, researchers have reported that the gut microbiota appears to be associated with cognitive function. Alterations in the microbiota of the gastrointestinal tract (dysbiosis) may be associated with inflammation and bowel disorders, and some studies suggest that the intestinal microbiota affects the behavior and brain biochemistry of mice [36, 37]. Damage of the gut tight junctions by microbiota changes has been reported to cause B-B-B damage and neuroinflammation in the brain tissue by releasing inflammatory cytokines from the gut into the blood. In addition, neurotrophic factors such as BDNF and several neurotransmitters such as brain serotonin, norepinephrine, and dopamine are produced as metabolites by the gut microbiota; thus, dysbiosis may cause cognitive decline [37]. According to Xu *et al*. (2020) [38], Spearman’s correlation analysis between cognitive ability and the gut microbiota revealed three negative correlations (*Oscillibacter*, *Mucispirillum*, and *Alistipes*) and four positive correlations (*Lactobacillus*, *Desulfovibrio*, *Bifidobacterium*, and *Saccharibacteria_ genera_incertae_sedis*) with spatial learning and memory ability at the genus level. Therefore, the regulation of microbiota could alleviate cognitive dysfunction.

Among the various gut metabolites, SCFAs produced by gut microbiota fermentation in the colon are important beneficial metabolites, which have been controlled to improve colonic tight junction integrity and systemic health [39]. The intake of β-glucan as a prebiotic attenuated a Aβ-induced cognitive impairment by modulating the gut microbiota, and the SCFAs (acetate and propionate) were also detected in the hippocampus of the brain, which were similar to fecal SCFAs [38]. Acetate and propionate could play important roles in the gut-brain axis. Acetate is involved in the insulin signaling pathway, and propionate is known to inhibit Aβ oligomer formation, B-B-B breakdown, and nonspecific inflammation [38]. On the other hand, butyrate is mainly used for gut homeostasis by downregulating indoleamine 2,3-dioxygenase-1 (IDO-1) expression in intestinal epithelial cells. IDO-1, which is an enzyme that catalyzes the oxidation of the indole moiety, plays a crucial role in obesity, atherosclerosis, vascular inflammation, and aneurysm by regulating the development and maintenance of the immune system [40]. In particular, IDO has been reported to catalyze the production of kynurenine from tryptophan in the brain, causing neuroinflammation, depression, and cognitive dysfunction by activating pre-inflammatory cytokines (IFN-γ, TNF-α, IL-1, and IL-6) [41]. These findings suggest that SCFAs produced in the gut may play an important role in memory function via the gut-brain axis. Based on our results (Fig. 4), the intake of the WEE could be helpful to restore the microbiota and produce SCFAs (acetate, propionate, and butyrate). Seaweed extracts have been reported to consist of components with prebiotic potential. The brown seaweed *Ecklonia radiata* stimulated beneficial bacteria (*Bifidobacterium* and *Lactobacillus*) and the production of SCFAs (butyrate) after in vitro fermentation for 24 h [42]. Taking together, the findings suggest that the gut microbiota may play physiological roles in not only gut regulation but also systemic immunity and cognitive function in the brain. The SCFAs produced from WEE intake may be beneficial gut metabolites for the treatment of cognitive decline through the gut-brain axis.

To determine whether the WEE had a protective effect on mice with PM_2.5_-induced cognitive dysfunction via the gut-brain axis, kynurenine metabolites were analyzed in the colon, blood plasma, and brain. The results indicated that the WEE effectively restored the level of KYNA, a neuroprotective metabolite, in the colon, blood plasma, and brain compared with the level in the PM_2.5_ exposed group (Fig. 5). Therefore, the WEE could improve cognitive function through the gut-brain axis. Among the gut-brain axis-related mechanisms, the kynurenine pathway is initiated by the degradation of tryptophan, which is mediated by IDO activation by inflammatory cytokines. Most of the degraded tryptophan is used for kynurenine production [12]. Notably, kynurenine is a molecule that can produce two different metabolites with opposing effects (*e.g.*, neuroprotective versus neurotoxic effects) [43]. KYNA is a metabolite with neuroprotective effects produced by kynurenine aminotransferases (KATs) [44]. It is possible that the WEE induced an increase in intestinal KYNA through the activation of KATs. Kynurenine and kynurenine metabolites produced in the intestines can be transported through blood vessels and affect various organs [45]. In particular, these metabolites can reach the brain directly by passing through the B-B-B [46]. Astrocytes are known to produce mainly KYNA, whereas microglia and macrophages produce mainly QUIN [47]. Chronic stress and inflammation in the periphery can cause microglial activation, which induces the production of QUIN and disrupts the balance between QUIN and KYNA. Based on our findings, a decreased KYNA level may function as a biomarker of cognitive dysfunction caused by PM_2.5_ in the microbiota-gut-brain axis, and the WEE may be used as a therapeutic that effectively protects against PM_2.5_ induced systemic damage by regulating KYNA production.

QUIN, a neurotoxic metabolite produced by the kynurenine pathway, was not detected in the brain and colon. However, the level of QUIN was increased in the blood plasma, indicating the systemic toxic effect of PM_2.5_ (Fig. 5). The administration of the WEE effectively restored the level of QUIN in the blood plasma. Although the correlation of PM_2.5_ with gut health has not been reported, our results suggest that PM_2.5_-induced gut dysfunction may be related to organ damage due to the release of QUIN into the blood. Recently, it has been reported that the regulation of the kynurenine pathway can not only improve cognitive function but also prevent systemic diseases [48, 49]. In the brain, QUIN causes neuronal cell death as a competitive agonist of N-methyl-D-aspartic acid receptor through excessive Ca^2+^ discharge, excitotoxic neurodegenerative changes, and lipid peroxidation [49]. Therefore, the development of KYNA analogs or synthesis-related inhibitors of QUIN is being considered as a potential therapeutic approach [50, 51]. The curcumin-piperine complex has been observed to enhance cognitive function in QUIN-induced neurodegenerative rat models by regulating inflammation (TNF-α and IL-1β), oxidative stress (lipid peroxidation, nitrite, and GSH), catechol amines, and GABA [49]. Essential fatty acid-rich diets could significantly prevent neurodegenerative diseases caused by QUIN in mice through modulation of GABA levels and peroxisome proliferator-activated receptor (PPAR)-γ expression [52]. Recent studies suggest that phytochemicals associated with antioxidant/inflammatory effects may regulate QUIN-induced oxidative damage in several neurological diseases [53].

Unfortunately, kynurenine pathway-related specific bacteria have not yet been reported. However, a recent study demonstrated the correlation between the kynurenine pathway and gut microbiota [54]. Microbiota-rich mice changed kynurenine pathway-related metabolites, in contrast to germ-free mice. In addition, other studies have reported that inflammation and TLRs activation was closely associated with the kynurenine pathway [9, 54]. In the brain of LPS-stimulated mice, an increase in KMO related to the production of QUNA was observed, not KAT-II as an enzyme related to the production of KYNA [55, 56]. Thus, receptors of pro-inflammatory cytokines (IFN-γ, IL-1β, and TNF) and TLR4 activated the kynurenine pathway by initiating TRP degradation towards KYN through an increase in IDO expression [55]. Indeed, KYNA decreased in the colon, blood plasma, and brains, while QUNA increased in blood plasma with PM_2.5_ exposure (Fig. 5). And the intake of WEE effectively regulated the KYNA and QUNA levels. The production of gut beneficial metabolites (SCFAs and KYNA) through the regulation of gut bacteria by fermentation of phlorotannins and fucoidan could be a possible mechnism for the health benefit effects of WEE. Another possible mechanism could be the regulation of TLR4-initiated inflammatory reaction by PM_2.5_. Taken together, our findings indicated that the administration of the WEE markedly ameliorated alterations in the microbiota and gut metabolites caused by PM_2.5_. In particular, KYNA as a neuroprotective metabolite could affect cognitive function through the microbiota-gut-brain axis. A conceptual model of the newly proposed kynurenine pathway could explain the health benefit effects of the WEE on PM_2.5_-induced systemic damages.

This study evaluated the ameliorating effect of *E. cava* on PM_2.5_-induced cognitive dysfunction via the gut-brain axis. The intake of the WEE effectively restored gut function and alleviated PM_2.5_ induced changes in the microbiota and associated metabolites. WEE intake also restored the gut microbiota by increasing the abundance of *Lactobacillus* (Family: Lactobacillaceae) and decreasing the abundance of *Alistipes* (Family: Rikenellaceae). In the gut-brain axis, the WEE induced the production of beneficial gut metabolites such as SCFAs and KYNA. Therefore, the findings demonstrated that the WEE could improve health benefit effects by producing metabolites (SCFAs and KYNA) through regulation of the microbiota.

## Figures and Tables

**Table 1:** Multiple reaction monitoring (MRM) conditions in the positive ESI mode.

Analyte	Precursor ion (m/z)	Product ion (*m*/*z*)	Collision energy (eV)
Tryptophan (TYP)	188.06	118.06	30
Kynurenine (KYN)	146.06	91.05	30
Kynurenic acid (KYNA)	162.05	116.04	30
Quinolinic acid (QUIN)	168.1	151.1	20
